# Content characteristics and information quality of flap transplantation surgery videos on TikTok and Bilibili: A cross-sectional study

**DOI:** 10.1097/MD.0000000000050873

**Published:** 2026-09-25

**Authors:** Qingyu Chen, Kaidi Zhao, Zhixuan Zhang, Yanqi Li

**Affiliations:** a The Second School of Medicine, Wenzhou Medical University, Wenzhou, Zhejiang, China; b Department of Dermatology, Xi’an Jiaotong University Second Affiliated Hospital, Xi’an, Shaanxi, China; c Department of Plastic Surgery, Emergency General Hospital, No. 29 XiBaHeNanLi, Chaoyang District, Beijing, China.

**Keywords:** Bilibili, flap transplantation, health information quality, reconstructive surgery, TikTok

## Abstract

Flap transplantation is a crucial reconstructive surgical technique, yet public understanding of its principles and postoperative management remains limited. With the rapid rise of short-form video platforms, social media has become an important medium for disseminating medical information. This study aimed to evaluate the content characteristics, educational quality, and reliability of flap transplantation surgery videos on TikTok and Bilibili. A cross-sectional content analysis was conducted to evaluate short videos related to flap transplantation surgery on TikTok and Bilibili. Videos were retrieved on September 25, 2025, using the Chinese keyword “皮瓣移植手术” (“flap transplantation surgery”), and searches were performed on the same day to reduce algorithm-related bias. After excluding duplicate, irrelevant, and commercial content, 236 videos were included. Two independent researchers extracted and verified data on video characteristics and engagement metrics. Educational content and information quality were assessed using the Global Quality Scale (GQS), modified DISCERN (mDISCERN), and Journal of the American Medical Association (JAMA) criteria. Statistical analyses were performed using Statistical Package for the Social Sciences version 27.0 with the Mann–Whitney *U* test, Kruskal–Wallis test, and Spearman correlation. Statistical significance was set at *P* < .05. Overall, the videos demonstrated moderate quality, with median scores of 3.00 (interquartile range 3.00–4.00) for GQS, 3.00 (2.00–3.00) for mDISCERN, and 3.00 (3.00–4.00) for JAMA. TikTok videos were significantly shorter (52.50 seconds vs 162.00 seconds, *P* < .001) but achieved higher GQS, mDISCERN, and JAMA scores (*P* < .001) compared with Bilibili. Specialist physicians accounted for 69.1% of uploaders and produced videos with significantly higher quality than science communicators or institutional/patient accounts (all pairwise *P* < .05). Engagement indicators such as likes and shares were strongly correlated with each other (up to *r* = 0.92) but not with video quality (*P* > .05). TikTok and Bilibili play an increasing role in disseminating surgical knowledge about flap transplantation. Although TikTok videos were shorter, they demonstrated higher structural clarity and informational reliability. Videos uploaded by physicians showed the highest accuracy and transparency, while nonprofessional content exhibited lower educational value. These findings highlight the need for improved content regulation, professional participation, and evidence-based design to enhance the educational impact of medical short videos.

## 1. Introduction

Flap transplantation is a key reconstructive surgical technique widely used to manage traumatic soft-tissue defects, repair tissues after tumor excision, and treat chronic wounds.^[1,2]^ Although advances in microsurgery have improved success rates to over 95 percent, the procedure remains technically demanding and requires accurate anatomical assessment, meticulous vascular anastomosis, and close postoperative monitoring.^[3–8]^ Because flap viability and recovery are highly dependent on postoperative care, patients’ understanding of surgical risks, procedural steps, and rehabilitation is essential for improving compliance and clinical outcomes.^[9,10]^

With the rapid expansion of digital media, short-video platforms such as TikTok and Bilibili have become major sources of medical information for the public.^[11–13]^ These platforms allow rapid and highly visual dissemination of surgical content, yet numerous studies have shown that health-related videos often contain incomplete, oversimplified, or unverified information.^[14–16]^ Recent research in digital health literacy and infodemiology has also demonstrated that social media platforms significantly shape public understanding of medical information across various health domains, including infectious diseases, dermatology, and oral health.^[17,18]^ These findings indicate that the variability observed in surgical video quality reflects broader patterns seen across online medical information ecosystems. While previous research has assessed the quality of videos related to dermatology, cosmetic procedures, gastrointestinal diseases, and other medical topics, little is known about the accuracy, completeness, and educational value of flap transplantation surgery videos.^[19–21]^ In addition, no study has systematically compared platform-level differences or examined whether user engagement indicators reflect actual information quality.

To address these gaps, the present study conducted a cross-sectional evaluation of flap transplantation surgery videos on TikTok and Bilibili. We assessed the educational completeness, reliability, and scientific quality of the videos using standardized instruments, compared differences across platforms and uploader categories, and examined correlations between content quality and engagement metrics. By identifying current strengths and weaknesses in flap transplantation education on Chinese short-video platforms, this study aims to provide evidence-based insights for improving the accuracy, accessibility, and educational value of surgical content disseminated to the public.

## 2. Methods

### 2.1. Study design

This study was a quantitative cross-sectional observational content analysis of short videos related to flap transplantation surgery on TikTok (Douyin) and Bilibili. The study was designed and reported in accordance with the Strengthening the Reporting of Observational Studies in Epidemiology guidelines for observational research.

### 2.2. Search strategy and setting

Videos were retrieved from TikTok and Bilibili on September 25, 2025, using the Chinese keyword “皮瓣移植手术” (“flap transplantation surgery”). To reduce algorithm-related personalization bias, searches were conducted on the same day on both platforms and performed through the default recommendation interface without logging into personal accounts or applying user-specific filters. Only Chinese-language videos were considered, consistent with the scope of Chinese short-video platforms.

### 2.3. Sampling and sample size

A consecutive sampling strategy was applied by screening videos in the order presented in the search results. Initially, 150 videos were retrieved from each platform. No formal sample size calculation was performed because consecutive sampling of the top-ranked search results is a commonly adopted approach in social media content analysis, where the platform-ranked output determines the analytic sample.

### 2.4. Eligibility criteria

Videos were included if they primarily addressed flap transplantation surgery, including its principles, procedures, perioperative management, complications, or related medical knowledge; if they were presented in Chinese with a duration of at least 10 seconds; and if the audiovisual content was clear and interpretable. Videos were excluded if they were irrelevant to flap transplantation (e.g., content focused solely on general cosmetic procedures or non-flap reconstructive surgery), if they had explicit commercial intent (defined as content primarily promoting hospitals, clinics, products, or services rather than providing educational information), if they were duplicates or reedited versions of the same original video, or if key information was missing due to playback errors or incomplete presentation.

### 2.5. Data extraction and verification

Each eligible video was treated as an independent unit of analysis. For each video, the uniform resource locator, title, upload date, uploader name, platform, video duration, and engagement metrics (views, likes, comments, favorites, and shares) were recorded. Two independent researchers extracted the data and cross-checked all entries; disagreements were resolved by discussion, and the final dataset was verified by a third researcher to ensure completeness and accuracy.

### 2.6. Exposure variables and outcome measures

The primary exposure variables were platform type (TikTok vs Bilibili) and uploader category. Uploaders were categorized as specialist physicians (licensed medical practitioners with clearly identifiable professional status), science communicators (nonphysician creators primarily disseminating medical or health information), and individuals or institutions (including patients, institutional accounts, and promotional or brand accounts).

The primary outcomes were video quality and reliability assessed using 3 established instruments: the Global Quality Scale (GQS; 1–5 points) for overall educational quality and usefulness^[22]^ (Table 1); the modified DISCERN (mDISCERN; 0–5 points) for reliability and transparency of medical information^[23]^ (Table 2); and the Journal of the American Medical Association (JAMA) benchmark criteria (0–4 points) assessing authorship, attribution, disclosure, and currency^[24]^ (Table 3). Secondary outcomes included engagement metrics and educational content coverage. Educational content was evaluated across 6 dimensions (general introduction, preoperative preparation, surgical procedure, intraoperative risks/complications, postoperative care/recovery, and indications/contraindications) using a 3-level coding scheme (0 not mentioned, 1 partially covered, 2 comprehensively and accurately covered).

**Table 1:** The GQS quality criteria.

Item features	Points
Poor quality; poor flow of the videos; most information missing; not at all useful for patients	1
Generally poor quality; some information listed, but many important topics missing; of very limited use to patients	2
Moderate quality; suboptimal flow; some important adequately discussed, but other information poorly discussed; somewhat useful for patients	3
Good quality and generally good flow; most of the relevant information listed, but some topics not covered; useful for patients	4
Excellent quality and flow; very useful for patients	5

GQS = Global Quality Scale.

**Table 2:** The mDISCERN quality criteria.

Reliability Score
1. Is the video clear, concise, and understandable?
2. Are valid sources cited?
3. Is the content presented balanced and unbiased?
4. Are additional sources of content listed for patient reference?
5. Are areas of uncertainty mentioned?

mDISCERN = modified DISCERN.

**Table 3:** The JAMA benchmark criteria.

Score*	Score component	
1 score	Authorship	Author and contributor credentials and their affiliations should be provided.
1 score	Attribution	Clearly lists all copyright information and states references and sources for content.
1 score	Currency	Initial date of posted content and subsequent updates to content should be provided.
1 score	Disclosure	Conflicts of interest, funding, sponsorship, advertising, support, and video ownership should be fully disclosed.

JAMA = Journal of the American Medical Association.

* The criteria for each aspect were scored separately, with 1 point for each criterion, for a total score of 4 points.

### 2.7. Quality assessment and inter-rater reliability

Two independent raters evaluated all videos using GQS, mDISCERN, and JAMA criteria. Discrepancies were resolved by consensus with a third reviewer. Inter-rater reliability was assessed using Cohen kappa, and internal consistency was evaluated using Cronbach alpha. The analysis demonstrated substantial agreement between raters across all 3 scales.

### 2.8. Statistical analysis

Statistical analyses were performed using Statistical Package for the Social Sciences version 27.0 (IBM Corp.). Continuous variables were summarized as median (interquartile range [IQR]), and categorical variables as frequency (percentage). Comparisons between TikTok and Bilibili were conducted using the Mann–Whitney *U* test. Differences among uploader categories were analyzed using the Kruskal–Wallis test, with post hoc pairwise comparisons performed using Dunn test with Bonferroni correction. Associations between engagement metrics and video quality scores were assessed using Spearman rank correlation. All tests were 2-tailed, and a *P* value < .05 was considered statistically significant; very small *P* values are reported as *P* < .001.

### 2.9. Ethics

This study analyzed publicly available and non-identifiable video content. No human participants, patient data, or identifiable personal information were involved; therefore, institutional review board approval was not required in accordance with institutional policy. The study endpoint was defined as completion of coding and quality assessment for all eligible videos.

## 3. Results

### 3.1. General characteristics, platform distribution, and uploader composition

A total of 300 short videos related to “皮瓣移植手术” were initially retrieved, including 150 from TikTok and 150 from Bilibili. After screening, 6 irrelevant and 4 promotional videos were excluded from TikTok, while 38 irrelevant, 10 promotional, and 6 duplicate videos were removed from Bilibili. Ultimately, 140 TikTok videos and 96 Bilibili videos were included for analysis, yielding a total of 236 videos (Fig. 1). The median video length was 76.50 seconds (IQR 37.00–162.00). The median numbers of likes, comments, favorites, and shares were 265.50 (48.00–931.00), 25.00 (2.00–77.00), 131.00 (16.75–365.50), and 69.00 (10.00–198.00), respectively (Table 4). Overall, video quality was moderate, with median GQS, mDISCERN, and JAMA scores all at 3.00 (IQRs: 3.00–4.00, 2.00–3.00, and 3.00–4.00, respectively).

**Table 4:** Descriptive characteristics of included videos.

Variables	Total (n = 236)
General information	
Video length, M (Q_1_, Q_3_)	76.50 (37.00, 162.00)
Likes, M (Q_1_, Q_3_)	265.50 (48.00, 931.00)
Collections, M (Q_1_, Q_3_)	131.00 (16.75, 365.50)
Comments, M (Q_1_, Q_3_)	25.00 (2.00, 77.00)
Shares, M (Q_1_, Q_3_)	69.00 (10.00, 198.00)
Video quality	
GQS score, M (Q_1_, Q_3_)	3.00 (3.00, 4.00)
mDISCERN sore, M (Q_1_, Q_3_)	3.00 (2.00, 3.00)
JAMA score, M (Q_1_, Q_3_)	3.00 (3.00, 4.00)
Video content	
General introduction, M (Q_1_, Q_3_)	0.00 (0.00, 1.00)
Preoperative preparation, M (Q_1_, Q_3_)	1.00 (0.00, 1.00)
Surgical procedure, M (Q_1_, Q_3_)	1.00 (0.00, 1.00)
Intraoperative risks and potential complications, M (Q_1_, Q_3_)	0.00 (0.00, 0.00)
Postoperative care and recovery, M (Q_1_, Q_3_)	0.00 (0.00, 1.00)
Indications and contraindications, M (Q_1_, Q_3_)	1.00 (0.00, 1.00)

GQS = Global Quality Scale, JAMA = Journal of the American Medical Association, M = median, mDISCERN = modified DISCERN, n = number of videos, Q_1_ = first quartile, Q_3_ = third quartile.

**Figure 1.**
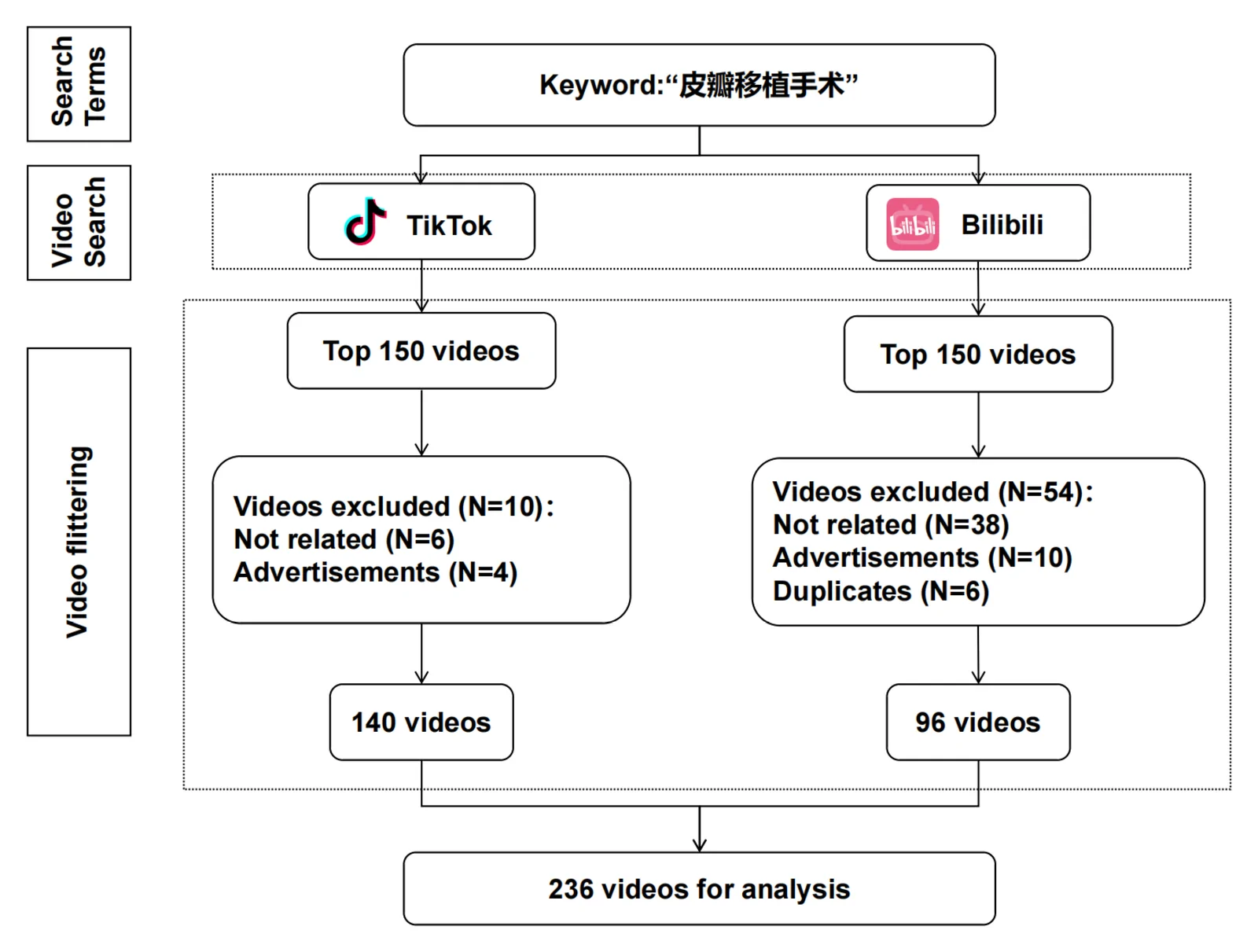
Flowchart of video screening and inclusion process for TikTok (Douyin) and Bilibili videos related to flap transplantation surgery, following predefined inclusion and exclusion criteria.

Among the videos, 140 (59.3%) were from TikTok and 96 (40.7%) from Bilibili (Fig. 2A). In terms of temporal trends, the number of videos on both platforms showed an overall upward trajectory, with a marked increase in recent years (Fig. 2B). Specialist physicians accounted for the majority of uploaders (69.1%), followed by science communicators (21.2%) and individuals or organizations (9.8%) (Fig. 2C). Figure 2D shows that specialist physicians comprised the largest proportion of TikTok uploaders (69%), whereas science communicators were more prevalent on Bilibili (42%), and individual or institutional uploaders accounted for smaller proportions on both platforms (7% vs 9%).

**Figure 2.**
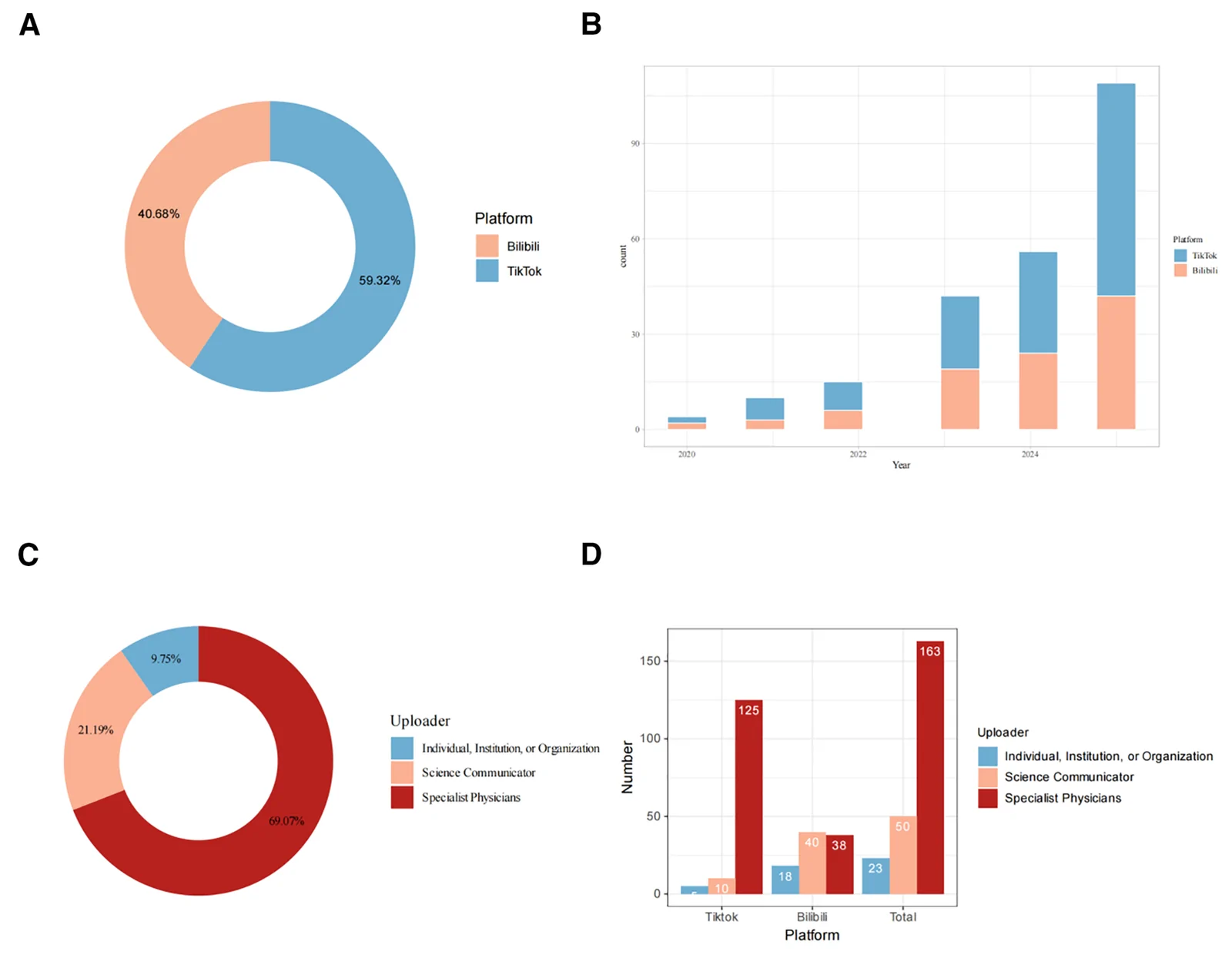
Characteristics of included videos on TikTok and Bilibili: (A) Platform distribution of included videos; (B) annual trend of video uploads; (C) overall composition of uploader categories; (D) distribution of uploader types on TikTok and Bilibili.

### 3.2. Video content characteristics

The video content could be categorized into 6 major domains: general introduction, preoperative preparation, surgical procedure, intraoperative risks and complications, postoperative care and recovery, and indications or contraindications (Table 4). Most videos focused on demonstrating the surgical procedure (median 1.00, IQR 0.00–1.00) and preoperative preparation (median 1.00, IQR 0.00–1.00), while information on intraoperative risks and complications (median 0.00, IQR 0.00–0.00) and postoperative care and recovery (median 0.00, IQR 0.00–1.00) was notably scarce. Only a small proportion of videos included information on surgical indications and contraindications (median 1.00, IQR 0.00–1.00).

From the perspective of content type (Fig. 3), TikTok videos primarily featured case demonstrations (48%) and professional education (34%), emphasizing clinical procedures and visual presentation. In contrast, Bilibili videos were more oriented toward scientific explanation (41%) and experience sharing (22%), focusing on theoretical principles and postoperative care in a more narrative or popular-science style.

**Figure 3.**
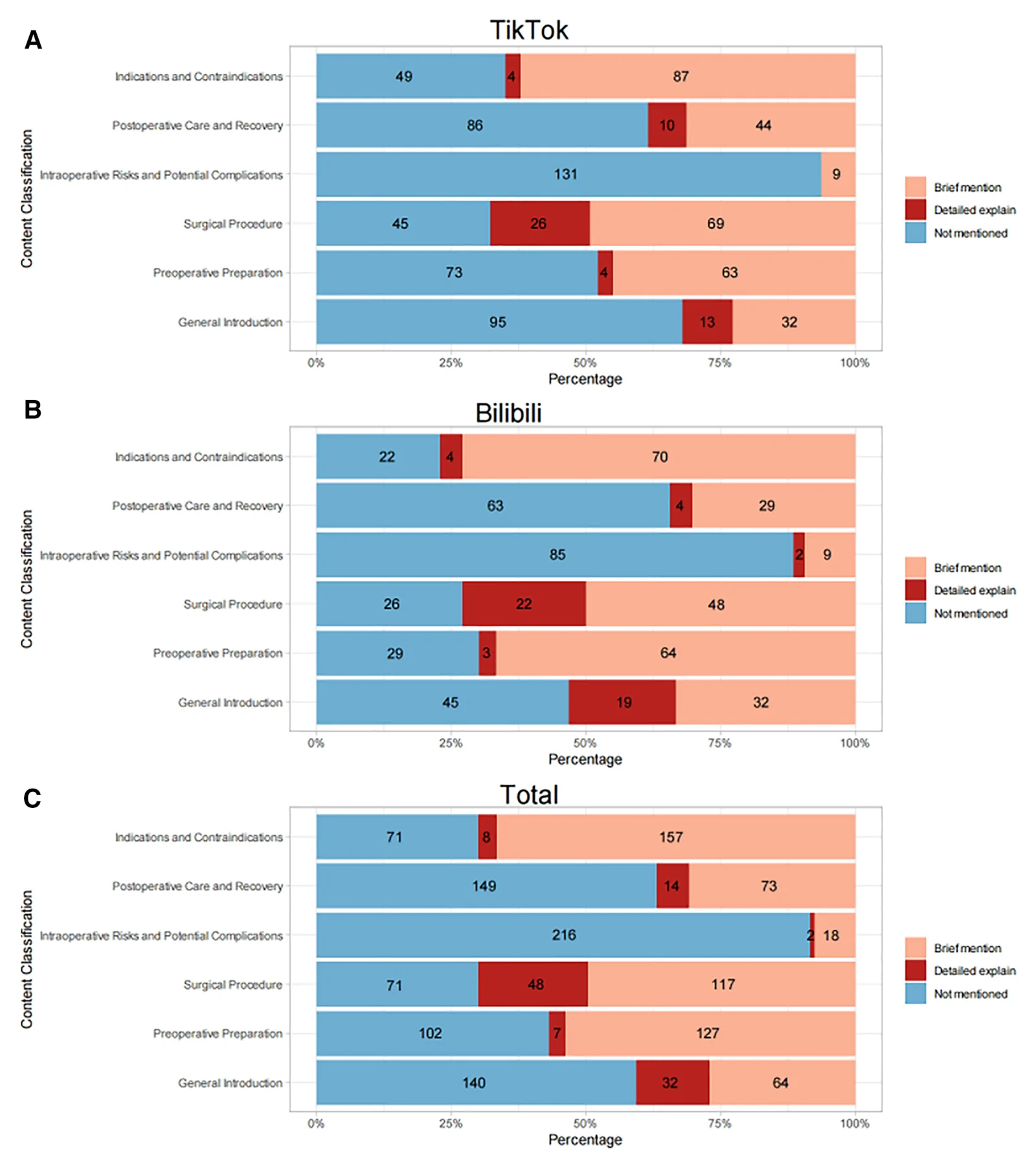
Distribution of educational content coverage across 6 predefined dimensions (general introduction, preoperative preparation, surgical procedure, intraoperative risks/complications, postoperative care/recovery, and indications/contraindications): (A) TikTok videos; (B) Bilibili videos; (C) overall distribution across both platforms.

### 3.3. Comparison of video quality and reliability between platforms

Significant differences were observed between TikTok and Bilibili videos across multiple characteristics and quality indicators. Bilibili videos were significantly longer (162.00 seconds vs 52.50 seconds; *P* < .001), indicating more comprehensive content, whereas TikTok videos received a higher number of likes (296.00 vs 234.50; *P* = .033) and shares (80.50 vs 53.00; *P* = .039). No significant differences were found in the number of favorites or comments between the 2 platforms (*P* > .05). Regarding quality metrics, TikTok outperformed Bilibili across all 3 scoring systems (Fig. 4, Table 5). The median GQS score for TikTok was 3.00 (IQR, 3.00–4.00) compared with 3.00 (IQR, 2.00–3.00) for Bilibili (*P* < .001). The mDISCERN score for TikTok was 3.00 (IQR, 3.00–3.00) versus 2.50 (IQR, 2.00–3.00) for Bilibili (*P* < .001), and the JAMA score was 4.00 (IQR, 3.00–4.00) for TikTok compared with 3.00 (IQR, 2.00–4.00) for Bilibili (*P* = .001). Although TikTok primarily features short videos, its content was more concise and structurally coherent, demonstrating higher overall educational quality and informational reliability.

**Table 5:** Comparison of video characteristics and quality scores between TikTok and Bilibili.

Variables	Total (n = 236)	Bilibili (n = 96)	TikTok (n = 140)	Statistic (*Z*)	*P*
Video length, M (Q_1_, Q_3_)	76.50 (37.00, 162.00)	162.00 (74.00, 589.75)	52.50 (30.00, 87.25)	−7.65	< .001
Likes, M (Q_1_, Q_3_)	265.50 (48.00, 931.00)	234.50 (16.50, 798.00)	296.00 (89.00, 1011.75)	−2.13	.033
Collections, M (Q_1_, Q_3_)	131.00 (16.75, 365.50)	162.00 (10.75, 389.00)	94.00 (22.75, 345.25)	−0.36	.718
Comments, M (Q_1_, Q_3_)	25.00 (2.00, 77.00)	20.50 (0.00, 75.50)	27.00 (6.00, 81.50)	−1.88	.060
Shares, M (Q_1_, Q_3_)	69.00 (10.00, 198.00)	53.00 (7.00, 162.00)	80.50 (16.50, 252.00)	−2.07	.039
GQS score, M (Q_1_, Q_3_)	3.00 (3.00, 4.00)	3.00 (2.00, 3.00)	3.00 (3.00, 4.00)	−3.64	< .001
Mdiscern Score, M (Q_1_, Q_3_)	3.00 (2.00, 3.00)	2.50 (2.00, 3.00)	3.00 (3.00, 3.00)	−5.01	< .001
JAMA score, M (Q_1_, Q_3_)	3.00 (3.00, 4.00)	3.00 (2.00, 4.00)	4.00 (3.00, 4.00)	−3.27	.001

*Z* is the test statistic.

GQS = Global Quality Scale, JAMA = Journal of the American Medical Association, M = median, mDISCERN = Modified DISCERN, n = number of videos, Q_1_ = first quartile, Q_3_ = third quartile.

**Figure 4.**
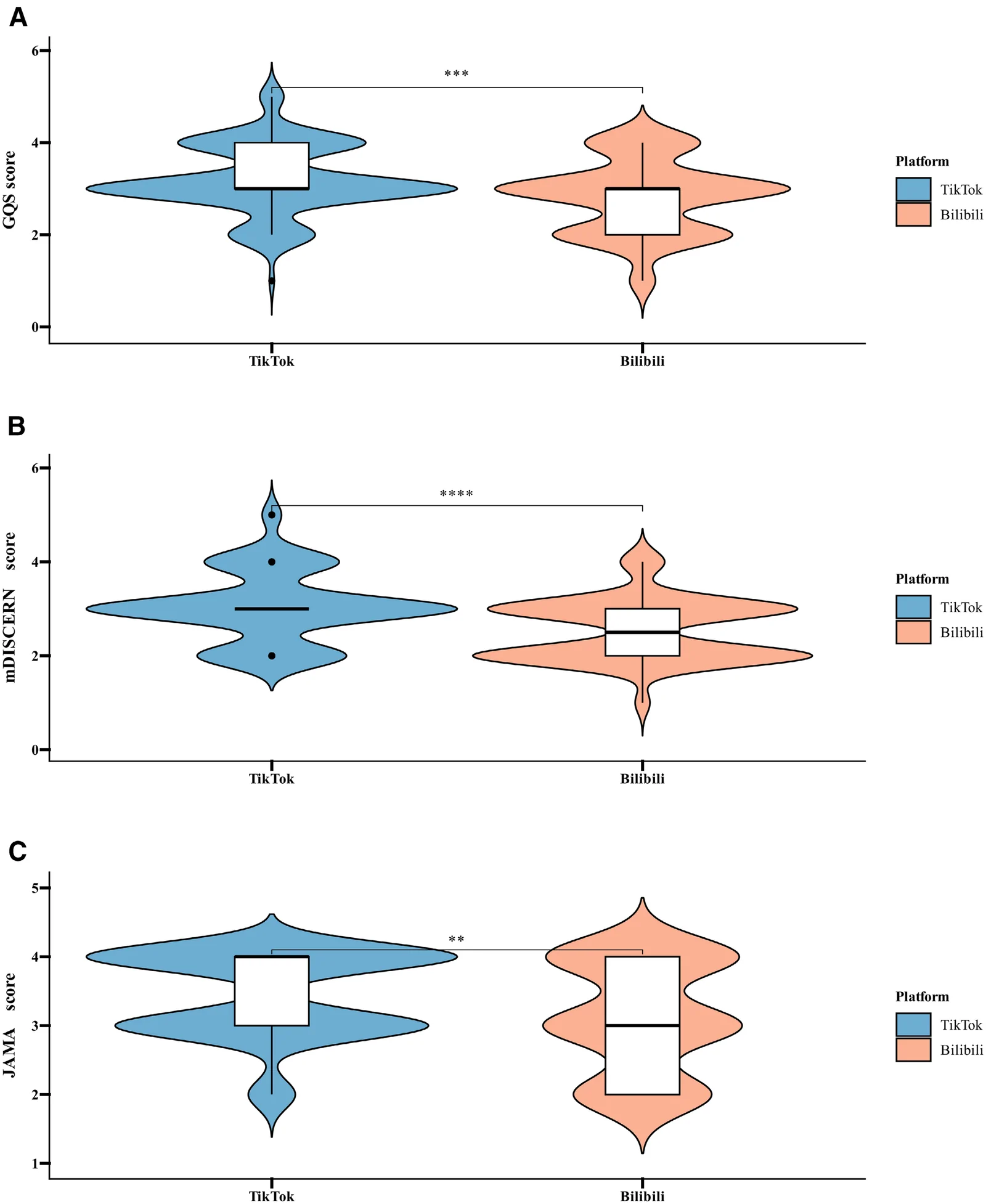
Comparison of video quality and reliability scores between TikTok and Bilibili: (A) GQS scores; (B) mDISCERN scores; (C) JAMA benchmark scores. Data are presented as median (IQR). Differences between platforms were assessed using the Mann–Whitney *U* test. GQS = Global Quality Scale, IQR = interquartile range, JAMA = Journal of the American Medical Association, mDISCERN = modified DISCERN. **P < .01; *** *P* < .001; **** *P* < .0001.

### 3.4. Comparison of video quality by uploader type

Video quality differed significantly across uploader categories (*P* < .001) (Table 6). As shown in Figure 5A, specialist physicians achieved the highest GQS scores (median, 3.00 [IQR, 3.00–4.00]), which were significantly higher than those of science communicators (3.00 [2.00–3.00]; *P* < .01) and individuals or institutions (2.00 [2.00–2.50]; *P* < .0001); the latter 2 groups also differed significantly (*P* < .001). For mDISCERN scores (Fig. 5B), physicians again ranked highest (3.00 [2.00–3.00]), significantly exceeding science communicators (3.00 [2.00–3.00]; *P* < .05) and individuals or institutions (2.00 [2.00–2.50]; *P* < .0001); the latter 2 groups also differed significantly (*P* < .01). Regarding JAMA scores (Fig. 5C), physicians achieved the highest ratings (4.00 [3.00–4.00]), surpassing both science communicators (3.00 [2.00–4.00]; *P* < .001) and individuals or institutions (3.00 [2.00–3.00]; *P* < .0001), whereas the difference between the latter 2 groups was not significant. Overall, videos uploaded by physicians exhibited superior medical accuracy, transparency, and reliability, whereas those produced by nonprofessional sources demonstrated comparatively lower quality, underscoring the strong influence of professional background on informational credibility.

**Table 6:** Comparison of video characteristics and quality scores by uploader type.

Variables	Total (n = 236)	Individual, institution, or organization (n = 23)	Science communicator (n = 50)	Specialist physicians (n = 163)	Statistic (*χ*^2^)	*P*
Video length, M (Q_1_, Q_3_)	76.50 (37.00, 162.00)	45.00 (30.50, 76.50)	325.00 (99.00, 712.00)	64.00 (35.00, 117.50)	54.68	< .001
Likes, M (Q_1_, Q_3_)	265.50 (48.00, 931.00)	366.00 (29.00, 1040.00)	55.00 (2.00, 432.00)	281.00 (90.00, 1022.00)	12.29	.002
Collections, M (Q_1_, Q_3_)	131.00 (16.75, 365.50)	95.00 (8.00, 204.00)	126.00 (9.00, 361.00)	135.00 (27.00, 390.00)	1.31	.521
Comments, M (Q_1_, Q_3_)	25.00 (2.00, 77.00)	77.00 (13.50, 758.00)	0.00 (0.00, 38.00)	30.00 (6.00, 73.50)	27.53	< .001
Shares, M (Q_1_, Q_3_)	69.00 (10.00, 198.00)	128.00 (5.50, 361.00)	49.00 (3.00, 128.00)	69.00 (16.00, 218.00)	6.31	.043
GQS score, M (Q_1_, Q_3_)	3.00 (3.00, 4.00)	2.00 (2.00, 2.50)	3.00 (2.00, 3.00)	3.00 (3.00, 4.00)	37.22	< .001
mDISCERN score, M (Q_1_, Q_3_)	3.00 (2.00, 3.00)	2.00 (2.00, 2.50)	3.00 (2.00, 3.00)	3.00 (2.00, 3.00)	24.97	< .001
JAMA score, M (Q_1_, Q_3_)	3.00 (3.00, 4.00)	3.00 (2.00, 3.00)	3.00 (2.00, 4.00)	4.00 (3.00, 4.00)	32.99	< .001

*χ*^2^ is the Kruskal–Wallis test statistic.

GQS = Global Quality Scale, JAMA = Journal of the American Medical Association, M = median, mDISCERN = Modified DISCERN, n = number of videos, Q_1_ = first quartile, Q_3_ = third quartile.

**Figure 5.**
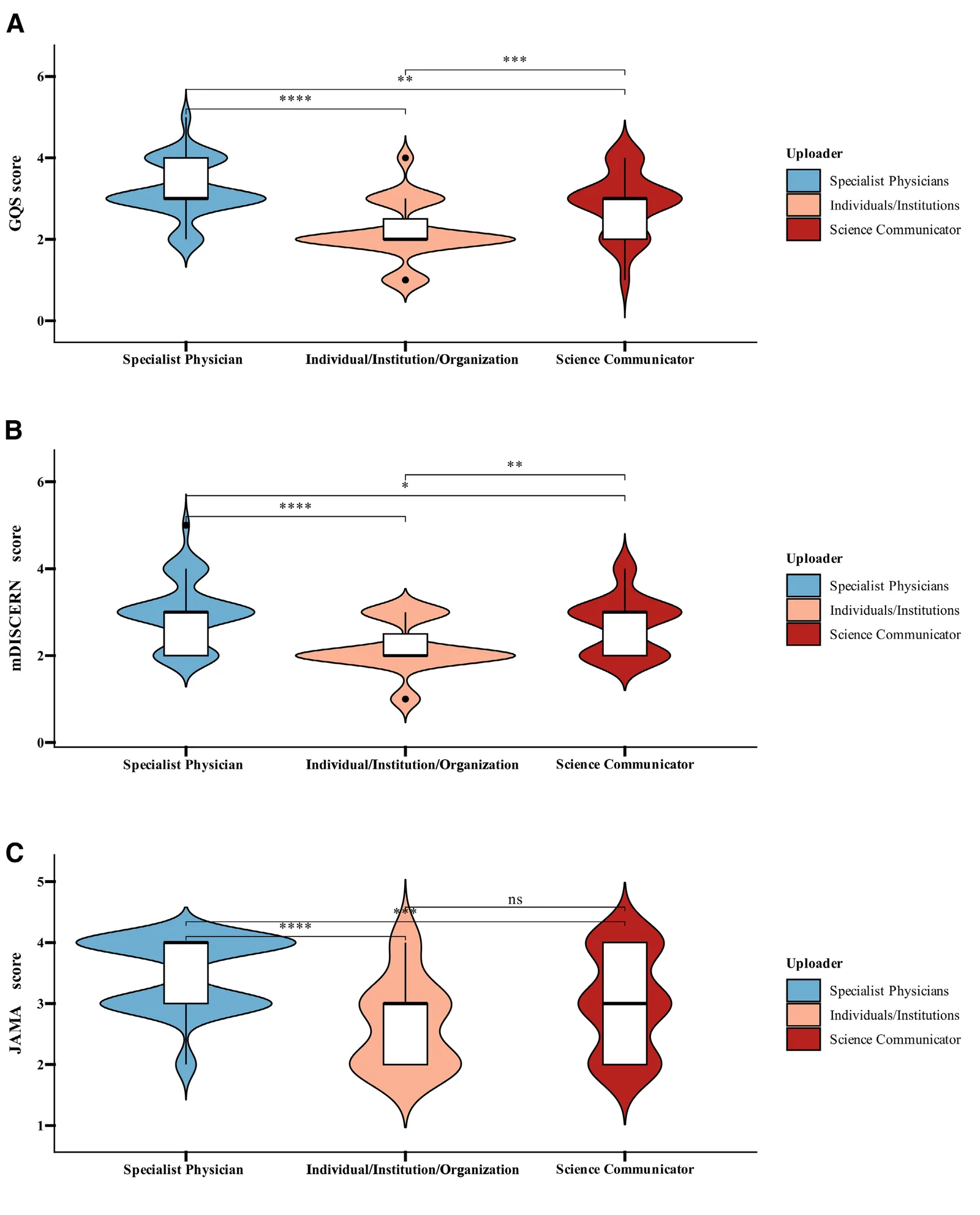
Comparison of video quality scores by uploader category (specialist physicians, science communicators, and individuals/institutions): (A) GQS scores; (B) mDISCERN scores; (C) JAMA scores. Data are presented as median (IQR). Differences among groups were analyzed using the Kruskal–Wallis test with Dunn post hoc pairwise comparisons and Bonferroni correction. GQS = Global Quality Scale, IQR = interquartile range, JAMA = Journal of the American Medical Association, mDISCERN = modified DISCERN, ns = not significant. *P* < .05; ** *P* < .01; *** *P* < .001; **** *P* < .0001.

### 3.5. Correlation between engagement metrics and video quality

Spearman correlation analysis revealed strong positive correlations among engagement indicators, particularly between likes and favorites (*r* = 0.92) and between comments and shares (*r* = 0.88) (Fig. 6). Other engagement metrics also exhibited moderate positive associations, indicating consistency in user interaction behaviors. Video length showed only weak correlations with engagement metrics (|*r*| < 0.1), suggesting that duration was not a key determinant of dissemination. No significant correlations were found between engagement indicators (likes, comments, favorites, shares) and video quality scores (GQS, mDISCERN, JAMA) (*P* > .05). Moderate positive correlations were observed among the 3 quality metrics themselves (GQS vs mDISCERN, *r* = 0.32; GQS vs JAMA, *r* = 0.29), indicating consistency among these scales in assessing the completeness and scientific rigor of video information.

**Figure 6.**
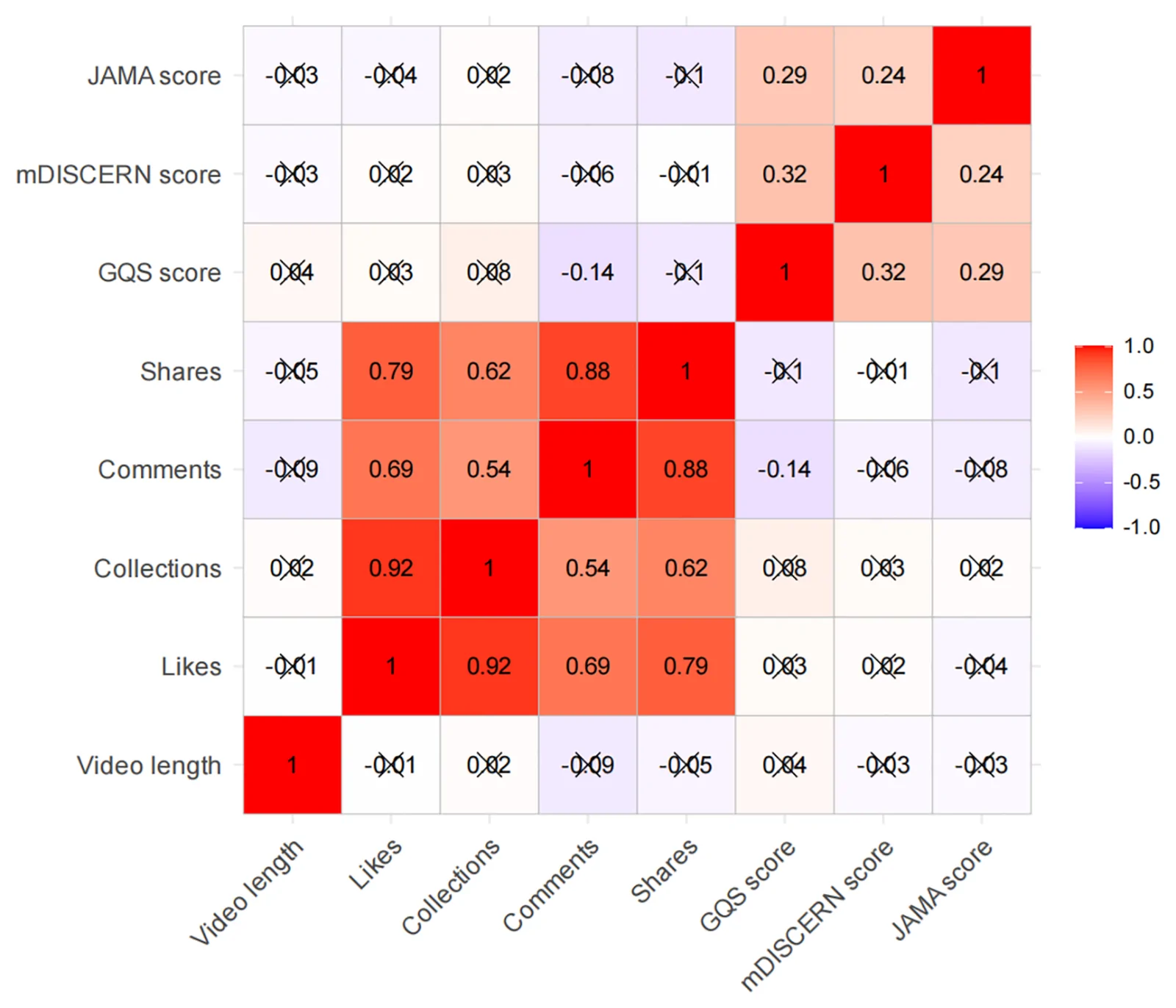
Spearman correlation heatmap illustrating relationships among engagement metrics (views, likes, comments, favorites, and shares) and video quality scores (GQS, mDISCERN, and JAMA). Warmer colors indicate stronger positive correlations, and cooler colors indicate weaker or negative correlations. GQS = Global Quality Scale, JAMA = Journal of the American Medical Association, mDISCERN = modified DISCERN.

## 4. Discussion

### 4.1. Main findings

In this cross-sectional analysis of short videos related to flap transplantation surgery on TikTok and Bilibili, we found that the overall quality of content was moderate, with median GQS, mDISCERN, and JAMA scores around 3 across both platforms. Although TikTok videos were significantly shorter than those on Bilibili, they demonstrated higher structural clarity and greater informational reliability. Video quality varied substantially according to uploader type, with specialist physicians consistently achieving the highest scores across all 3 assessment tools, whereas content from science communicators and individual or institutional accounts showed lower educational value. Furthermore, while engagement metrics such as likes, comments, favorites, and shares were strongly correlated with each other, none of these indicators showed a significant association with video quality.

### 4.2. Comparison with other studies

Our findings align with a growing body of literature examining the quality of medical information on social media. Similar to studies evaluating dermatology, oral health, and plastic surgery content on short-video platforms, we observed marked variability in information quality and a tendency for videos to prioritize visually engaging demonstrations over comprehensive patient education.^[25–30]^

Previous analyses have also reported that short-form surgical and health-related videos frequently underrepresent risks, postoperative care, and evidence-based explanations, which is consistent with our observation that intraoperative complications and postoperative management were the least covered dimensions in flap transplantation videos.^[31,32]^

Consistent with prior research in dermatology and other medical domains, we found that videos created by healthcare professionals exhibited higher reliability and educational completeness than those produced by nonprofessional creators, suggesting that professional authorship is a critical determinant of information quality across diverse medical topics.^[33,34]^

Additionally, our finding that user engagement did not correlate with content quality echoes results from earlier studies on TikTok, Instagram, and YouTube, reinforcing the notion that popularity does not necessarily reflect accuracy or educational merit in online health communication.^[35–37]^

### 4.3. Implications and explanations

Several factors may explain the patterns observed in this study. The predominance of surgical demonstrations and the relative scarcity of content on postoperative care and complications likely reflect the visual-driven nature of short-video platforms, which tend to reward aesthetic appeal, dramatic surgical footage, and concise storytelling rather than comprehensive medical explanation. This pattern has been repeatedly described in analyses of aesthetic and plastic surgery content on TikTok.^[25,26,28]^

The superior performance of TikTok videos, despite their shorter duration, may be related to the platform’s emphasis on concise, structured, and highly edited content, which can facilitate clearer message delivery. In contrast, Bilibili’s more heterogeneous creator community and longer video format may allow for greater depth but also introduce more variability in accuracy and presentation quality, a trend also observed in comparative studies of TikTok and Bilibili for other medical topics.^[38]^

From a clinical perspective, the limited coverage of intraoperative risks and postoperative care is concerning because patient understanding of these elements is essential for informed decision-making, adherence to medical advice, and successful surgical outcomes.^[31,32]^ The disconnect between engagement and quality further suggests that platform algorithms and audience behavior may prioritize entertainment value over educational rigor. This pattern can be interpreted through the Elaboration Likelihood Model of persuasion; under conditions of limited attention typical of short-video consumption, users are more likely to rely on peripheral cues such as visual appeal and popularity indicators (likes and shares) rather than critically evaluating substantive medical content, which helps explain why engagement does not necessarily correspond to information quality.^[39]^

Collectively, these findings highlight the need for improved content regulation, greater professional participation, and more evidence-based design in medical short-video communication to ensure that public-facing surgical information is both engaging and scientifically sound, a concern repeatedly emphasized in digital health and social media research.^[33,39]^

### 4.4. Strengths and limitations

This study has several strengths. It provides a systematic, multi-platform comparison of flap transplantation surgery videos using 3 validated assessment tools (GQS, mDISCERN, and JAMA), offering a comprehensive evaluation of both educational quality and reliability. The use of independent dual coding, cross-verification, and inter-rater reliability testing further enhances the methodological rigor and reproducibility of our findings.

However, this study also has limitations. First, its cross-sectional design captures only a snapshot in time and does not account for temporal changes in content quality or platform policies. Second, although standardized scoring tools were used, some degree of subjectivity in video evaluation is unavoidable. Third, because our analysis was based solely on content characteristics, we did not directly measure audience comprehension, knowledge retention, or behavioral outcomes; therefore, our conclusions regarding educational implications are inferred rather than empirically validated. Future studies should incorporate audience-based or behavioral outcomes, such as comprehension, information retention, attitude change, or health-related behavioral intentions, to more rigorously evaluate the true educational effect of medical short videos. Finally, despite efforts to minimize personalization bias, the algorithm-driven nature of platform recommendations may still have influenced the sample of videos analyzed.

### 4.5. Conclusions, recommendations, and future directions

In conclusion, short videos on flap transplantation surgery exhibit overall moderate quality with substantial variation across platforms and uploader types. TikTok videos and physician-generated content demonstrated higher reliability and structural clarity, whereas nonprofessional content tended to be less comprehensive. Importantly, user engagement did not reflect informational quality, underscoring the limitations of popularity as a proxy for educational value, a pattern also documented in analyses of plastic surgery content on TikTok and Instagram.^[37]^

To improve the quality of online surgical information, we recommend greater involvement of medical professionals in short-video production, alongside collaboration with educators and media specialists to enhance clarity and accessibility. Platforms should consider implementing verification or quality-labeling mechanisms for medical content to guide users toward reliable sources, as suggested in studies of cervical cancer and fibromyalgia content on short-video platforms.^[33,34]^

Future research should incorporate audience-centered outcomes, such as comprehension, attitude change, and behavioral impact, to more accurately assess the true educational value of medical short videos, rather than relying solely on content metrics, as recommended in digital health scoping reviews.

## 5. Conclusion

Overall, short videos on flap transplantation surgery exhibit moderate informational quality with substantial variability across platforms and uploader types. TikTok videos and content produced by specialist physicians were more reliable and better structured than those from Bilibili and nonprofessional creators. Popularity, as reflected by user engagement, did not correspond to information quality. These findings highlight the need for greater professional participation and standardized guidelines for medical short-video communication.

## Author contributions

**Conceptualization:** Qingyu Chen.

**Data curation:** Kaidi Zhao.

**Formal analysis:** Qingyu Chen.

**Methodology:** Zhixuan Zhang.

**Project administration:** Yanqi Li.

**Software:** Zhixuan Zhang.

**Supervision:** Yanqi Li.

**Validation:** Kaidi Zhao.

**Visualization:** Qingyu Chen.

**Writing – original draft:** Qingyu Chen.

**Writing – review & editing:** Kaidi Zhao, Yanqi Li.
